# Exploring barriers to guideline implementation for prescription of surgical antibiotic prophylaxis in Nigeria

**DOI:** 10.1093/jacamr/dlac044

**Published:** 2022-04-19

**Authors:** Caroline E. Wood, Susanne Luedtke, Anwar Musah, Funmi Bammeke, Bamidele Mutiu, Rufus Ojewola, Olufemi Bankole, Adesoji Oludotun Ademuyiwa, Chibuzo Barbara Ekumankama, Folasade Ogunsola, Patrick Okonji, Eneyi E. Kpokiri, Theophilus Ayibanoah, Neni Aworabhi-Oki, Laura Shallcross, Andreea Molnar, Sue Wiseman, Andrew Hayward, Delphine Soriano, Georgiana Birjovanu, Carmen Lefevre, Olajumoke Olufemi, Patty Kostkova

**Affiliations:** 1 UCL IRDR Centre for Digital Public Health in Emergencies, University College London, London, UK; 2 Department of Sociology, University of Lagos, Lagos, Nigeria; 3 Department of Medical Microbiology, Lagos State University College of Medicine/Lagos State University Teaching Hospital, Lagos, Nigeria; 4 Urology Unit, Department of Surgery, College of Medicine University of Lagos/Lagos University Teaching Hospital, Lagos, Nigeria; 5 Neurosurgery Unit, Department of Surgery, College of Medicine University of Lagos/Lagos University Teaching Hospital, Lagos, Nigeria; 6 Paediatric Surgery Unit, Department of Surgery, College of Medicine University of Lagos/Lagos University Teaching Hospital, Lagos, Nigeria; 7 Department of Ophthalmology, Lagos State University Teaching Hospital, Lagos, Nigeria; 8 Faculty of Infectious and Tropical Diseases, London School of Hygiene and Tropical Medicine, London, UK; 9 Department of Pharmacy, Niger Delta University Teaching Hospital, Bayelsa State, Nigeria; 10 Department of Surgery, Niger Delta University Teaching Hospital, Bayelsa State, Nigeria; 11 Institute of Health Informatics, University College London, London, UK; 12 School of Software and Electrical Engineering, Swinburne University of Technology, Melbourne, Australia; 13 Institute of Epidemiology and Public Health, University College London, London, UK; 14 UCL Centre for Behaviour Change, University College London, London, UK

## Abstract

**Background:**

In Nigeria, the prescription of surgical antibiotic prophylaxis for prevention of surgical site infection tends to be driven by local policy rather than by published guidelines (e.g. WHO and Sanford).

**Objectives:**

To triangulate three datasets and understand key barriers to implementation using a behavioural science framework.

**Methods:**

Surgeons (*N *= 94) from three teaching hospitals in Nigeria participated in an online survey and in focus group discussions about barriers to implementation. The theoretical domains framework (TDF) was used to structure question items and interview schedules. A subgroup (*N *= 20) piloted a gamified decision support app over the course of 6 months and reported barriers at the point of care.

**Results:**

Knowledge of guidelines and intention to implement them in practice was high. Key barriers to implementation were related to environmental context and resources and concern over potential consequences of implementing recommendations within the Nigerian context applicable for similar settings in low-to-middle-income countries.

**Conclusions:**

The environmental context and limited resource setting of Nigerian hospitals currently presents a significant barrier to implementation of WHO and Sanford guidelines. Research and data collected from the local context must directly inform the writing of future international guidelines to increase rates of implementation.

## Introduction

Surgical site infection (SSI) is higher in low-to-middle-income countries such as Nigeria than in developed countries.^1^ Antibiotics are the most widely prescribed antimicrobial agents to treat SSIs. Inappropriate and irrational prescribing contributes to the escalating global problem of antimicrobial resistance (AMR).^2^ In Nigeria, there is already widespread resistance among enteric *Escherichia coli* particularly to penicillins, aminoglycosides, cephalosporins, chloramphenicol, tetracyclines and co-trimoxazole^3^ and a marked resistance to all drugs commonly prescribed for urinary tract infections.^4^ Without antimicrobials to prevent and treat infection, procedures from treatment and management of common infections through to major surgeries will become extremely high risk.

Half of prescribed antimicrobials in Africa are unnecessary or outside the recommendations of published guidance.^5^ Diversion away from recommendations for surgical antibiotic prophylaxis (SAP) (i.e. antibiotics administered shortly before, during or immediately after surgery) is common.^6,7^ Patients receive prolonged dosage for prophylaxis as a standard preventative against SSIs (despite the lack of scientific evidence supporting its benefits^1^) or SAP is prescribed for clean, non-invasive surgeries where there is no requirement.^8^ Antimicrobial prescribing stewardship (APS) programmes seek to educate and advocate use of prescribing guidelines.^9^ There are significant inadequacies in provision and implementation of APS programmes across Nigeria. Fadare and colleagues^10^ report that few hospitals have a formal organization structure and team responsible for APS. Of the 17 hospitals surveyed, 4 based their treatment recommendations on local resistance patterns, 2 had formal procedures for reviewing antibiotic prescriptions after 48 h and 1 routinely monitored antibiotic use. Choice of antibiotic is influenced by environmental factors such as pharmaceutical promotion, costs to patients, availability of antibiotics^11^ and social factors such as pressure to prescribe from patients and instruction from senior colleagues.^12^ Infrastructure supporting implementation of guidelines is absent with guidelines being weakly enforced, if at all.^13^ Inclination to follow policy is low, with less than half of doctors planning to refer to local policy and less than 10% stating they regularly used policy and guidelines.^11^

To increase the number of surgeons in Nigeria referring to guidelines, and to inform the ongoing development of effective countrywide APS programmes, work is needed to understand the environmental and social context of decision-making in surgery and how motivational and belief structures interact. The benefits of using behavioural science in this way to understand influences on behaviour, mechanisms of change and inform effective development of behaviour change interventions are widely known.^14,15^

Through a unique cross-disciplinary partnership connecting UK and Nigerian partners, the Gamified Antimicrobial Stewardship Decision Support App (GADSA) project aims to improve antibiotic stewardship amongst surgeons in Nigeria.^16,17,18^ GADSA is a mobile decision-support tool built on psychological principles and persuasive games techniques of using a mentor to deliver immediate positive and negative feedback on prescribing decisions, adopting ‘reinforced learning’ game techniques for users in low-to-middle-income countries.^19,20^ To our knowledge, the GADSA project is the first of its kind to integrate principles of behaviour change, gamification and customizable decision tree-based algorithm technology integrating published evidence-based guidance to deliver support at the point of care and drive down incidents of prescribing decisions misaligned with guidance. The development and evaluation of the GADSA app is described in full elsewhere^16^—see also S. Luedtke, C. E. Wood, A. Musah, F. Bammeke, B. Mutiu, R. Ojewola, O. Bankole, A. O. Ademuyiwa, C. B. Ekumankama, F. Ogunsola, P. Okonji, E. E. Kpokiri, T. Ayibanoah, N. Aworabhi-Oki, L. Shallcross, A. Molnar, S. Wiseman, A. Hayward, D. Soriano, G. Birjovanu, C. Lefevre, O. Olufemi and P. Kostkova (unpublished results), ‘Implementation of the Gamified Antimicrobial Decision Support App (GADSA) for surgeons in Nigeria: a hospital-based pilot study’—while the technical design project is covered in A. Molnar, A. Musah, S. Luedtke, C. E. Wood, F. Bammeke, B. Mutiu, R. Ojewola, O. Bankole, A. O. Ademuyiwa, C. B. Ekumankama, F. Ogunsola, P. Okonji, E. E. Kpokiri, T. Ayibanoah, N. Aworabhi-Oki, L. Shallcross, S. Wiseman, A. Hayward, D. Soriano, G. Birjovanu, C. Lefevre, O. Olufemi and P. Kostkova (unpublished results), ‘GADSA: persuasive Gamified Antimicrobial Stewardship Decision Support App for prescribing behaviour change in Nigeria’.

In this paper, we report findings from the first phase of the GADSA project which sought to understand prescribing in the Nigerian context and explore guideline implementation barriers. We explore attitudes and behavioural determinants in professionals working in three hospitals in Nigeria. We look at how these factors influence guideline informed prescription of SAP, specifically for elective surgeries to prevent SSI. We report a triangulation of data collected from three study phases: (i) an online survey; (i) a series of semi-structured focus group interviews; and (iii) a pilot of the GADSA app.

## Methods

### Setting

The setting was three tertiary teaching hospitals in Nigeria: Lagos University Teaching Hospital (LUTH) located in Idi-Araba, Surulere—a large modern hospital (761 beds); Lagos State University Teaching Hospital (LASUTH) in Ikeja, Lagos State—a younger, government-owned hospital (774 beds); and Niger Delta University Teaching Hospital (NDUTH)—much smaller and one of two tertiary hospitals in Bayelsa State, central to the Niger Delta region (200 beds). There is a newly established APS programme at LUTH and staff are still being taken through the protocols. NDUTH has recently established an APS team comprising clinical staff and an administrative member of staff. The team oversees procurement, documentations and monitors use of antimicrobials within the hospital.

### Participants

Participants were healthcare professionals (i.e. junior or senior residents, nurses, consultants or senior consultants) at one of the three sites, with responsibility for making SAP prescribing decisions. Participants were required to own or to have access to a smartphone with an Android operating system to participate in Phase 3. The project PI in Nigeria (F.O.) recruited senior consultants and their departmental teams through personal contacts at LUTH in April 2019. Two weeks later, two researchers (O.O., P.O.) visited teams on-site at LUTH and LASUTH to confirm participation. Participants at NDUTH were recruited in July by project Co-I (E.E.K.) and a senior pharmacist (T.A.).

### Theoretical framework

Two theory-based frameworks to structure our investigation; the COM-B model^21,22^ and the theoretical domains framework (TDF).^23,24^ The COM-B model provides a simple framework for understanding behaviour in which ‘capability’ (physical and psychological), ‘opportunity’ (physical and social) and ‘motivation’ (reflective and automatic) represent three conditions for a behaviour to occur. The TDF is a synthesis of behaviour change theories and can be used alongside the COM-B model to give a more fine-grained analysis of behaviour. Both the COM-B and TDF have been used effectively to identify influences on behaviour across a wide range of health professional behaviours including guideline appropriate prescribing (e.g.^25,26,27^).

### Methodology

There were three phases of data collection: (i) online survey, (ii) focus groups and (iii) in-app collected responses during piloting of the GASDA app.

#### Phase 1

Between April and July 2019, participants received a link (via e-mail or WhatsApp) to an online survey about SAP prescribing and experience of using guidelines. The 30 item survey was developed using the TDF as a guide. Survey items were refined through a process of discussion and consensus by the project team. Participants were asked to rate responses to each item using a Likert scale from 1 (strongly disagree) to 5 (strongly agree). Participants also indicated their place of work, age, gender, profession (junior/senior resident, consultant) and number of years in surgical specialism. Sixty-six (64 junior residents, 2 senior residents) completed the survey.

#### Phase 2

Four focus groups were held at LUTH and LASUTH (28 professionals) in April and June 2019 to further explore guideline appropriate prescribing. To increase participant numbers, phone calls and visits were made to individuals by the project PI (F.O.) and two researchers (O.O., P.O.). Groups lasted approximately 90 min and were facilitated by the fourth author (F.B.) and two researchers (O.O., P.O.). An interview guide based on the COM-B model with probing questions informed by domains from the TDF prompted discussion. Items included areas of interest (e.g. specific barriers, complexities of practice) arising from the online survey. In the last 30 min of the discussion, surgeons contributed to a co-design session for development of the GADSA app. Focus groups were audio-recorded and later transcribed and anonymized for analysis.

#### Phase 3

Between June and December 2019, surgeons were invited to participate in a 6-month pilot to use the GADSA app and record SAP prescriptions for all elective procedures. Sixty-five physicians consented, of whom 20 actively participated. A total of 343 prescriptions were recorded during the pilot. For SAP prescriptions where decisions did not match recommendations made by guidelines (*N *= 266; 77%), surgeons selected a reason for inappropriate prescribing from a list of frequently cited reasons or provided their own.

### Ethics

Ethical approvals from the UCL Ethics Committee and College of Medicine Ethical committee were obtained for all phases of this study prior to data being collected (ID: 11491/001). The purpose for each phase was described to participants and all provided consent before taking part. Data were anonymized prior to analysis using a combination of pseudonyms and numbers.

### Analysis

Surveys were exported from SurveyMonkey.com to IBM SPSS Statistics version 26 for analysis. Negatively scored items (e.g. ‘I find it difficult to follow the guidance because we often do not have access to the recommended SAPs’) were reverse-coded. Cronbach’s α was calculated for each domain to assess internal consistency of the items in each domain. Mean scores were calculated for each question item. Higher mean scores represented greater agreement with the statement. An overall mean score was calculated for each of the 12 domains represented in the survey. Focus group transcripts were coded independently by two researchers (C.E.W., S.L.) using the COM-B model and the TDF to structure themes identified. The researchers compared their coding after the third and the sixth transcripts to discuss discrepancies in coding and to reach consensus. Data collected by the GADSA app on reasons for diverting from guidelines in terms of SAP type and duration were exported into SPSS and the frequency with which each reason was reported was calculated.

## Results

Table 1 shows details of participants across each phase. Cronbach’s α values for the domains ranged from 0.67 (belief about capabilities) to 0.91 (behavioural regulation). The α value for the beliefs about capabilities domain (0.70) was considered adequate given the survey served as an exploratory analysis.

**Table 1.** Demographic characteristics of participants in each phase

Variable	Online survey (*N *= 66)		Focus groups (*N *= 28)		GADSA app (*N *= 20)	
	*n*	%	*n*	%	*n*	%
Gender						
Male	40	60.6	20	71.4	11	55.0
Female	24	36.4	8	28.6	9	45.0
Age (years)						
25–34	16	24.2	6	21.4	8	40.0
35–44	31	47.0	12	42.9	9	45.0
45–54	16	24.2	9	32.1	2	10.0
55–64	3	4.5	1	3.6	1	5.0
Profession						
Junior resident	64	97.0	—	—	10	50.0
Senior resident	2	3.0	10	35.7	6	30.0
Consultant	—	—	18	64.3	4	20.0
Experience (years)						
0–5	20	30.3	—	—	5	25.0
5–10	30	30.3	10	35.7	7	35.0
10–15	17	25.8	11	39.3	5	25.0
15+	9	13.6	7	25.0	3	15.0

Survey data, focus group data and reasons for diverting away from guidelines collected at the point of care by the GADSA app were well aligned. Table 2 presents a preliminary triangulation of these three datasets with further discussion of findings below.

**Table 2.** Illustrative quotes of barriers reported by participants assigned to COM-B components and theoretical domains

TDF domain	Response for survey items in domain (online survey), mean; SD^a^	Example quote(s) from focus group data	Reasons for diversion from guideline recommendations, as recorded in GASDA app by surgeons (*n*; %)^b^
Beliefs about consequences	4.32; 0.80	‘Most patients even leave the hospital before 24-hours, so we need to prolong the antibiotic more than 24 hours to secure them’ [P2, FG4]	Patient’s environment requires prolonged dose (56; 21.0%)
Intention	4.31; 0.71	‘I think it’s more for the Junior Cadre e.g. the house officers. Most Senior Cadre will be reluctant to change what they are already used to’ [P1, FG2]	No relevant reason currently exists in app
Knowledge	3.95; 0.69	‘Because of our peculiarities (in Nigeria), we have to do away with the guidelines and do what we think is the best in that situation because of the theatre environment’ [P3, FG6]	Patient’s environment requires prolonged dose (56; 21.0%)
Skills	3.77; 0.69	‘…while I don’t blame the residents, I think it’s based on the infection control training in the hospitals too…’ [P8, FG1]	No relevant reason currently exists in app
Social professional role and identity	3.71; 1.01	‘The challenge we have is that we are resident doctors and we follow whatever our consultant, our seniors, our boss tell us to follow’ [P1, FG5]	Following local practice (111; 41.7%)
		‘…most of our practice is based on what is past performance, broadly generation of training one to the other’ [P8, FG1]	Previous experience with this procedure (20; 7.5%)
			Advised by a colleague (2; 0.8%)
Beliefs about capabilities	3.59; 0.62	‘Guidelines are recommendations (…) if in your judgement, you really think that the patient should do better with other medication or strategies outside the guideline, I don’t think you should be cast in iron’ [P2, FG1]	No relevant reason currently exists in app
Reinforcement	3.28; 0.82	‘…there is no policy. There’s nobody cross-checking anything. It’s not documented [unclear] prescribing pattern, protocols…’ [P8, FG1]	No relevant reason currently exists in app
Memory, attention and decision processes	2.53; 0.87	Research needs to be carried out here (in Nigeria) based on our own circumstances and see the results. It needs to be modified to our own situation’ [P2, FG2]	Previous experience with this procedure (20; 7.5%)
Environmental context and resources	2.47; 0.70	‘In this part of the world, you may have to prolong the use of antibiotic because you are not sure of the environment. Our environment here is not as clean as that of the country where the guideline came from, so following this kind of guideline can be disastrous’ [P1, FG2]	Patient is at high risk for developing SSI (59; 22.2%)
		‘Some patients are not comfortable not getting some antibiotics after surgery. If you operate a patient today and you don’t give antibiotics, the patient will say “no…no… you didn’t give me drugs after surgery” whereas the guideline says just this surgery, but the patient will not agree. They insist you must give us something to use, and if you don’t give them, the patient will go and meet another person’ [P2, FG5]	Patient has complications requiring prolonged dose (6; 2.3%)
			Patient’s environment requires prolonged dose (56; 21.0%)
Emotion	2.17; 0.88	Not coded in focus group data	No relevant reason currently exists in app

a Rating scale for responses to survey items was from 1 (strongly disagree) to 5 (strongly agree); negatively phrased items were recoded so for all items, the closer a mean score was to 5, the more surgeons agreed with the statements.

b Out of a total 266 prescriptions diverting from guidelines.

Surgeons reported familiarity with WHO guidelines, but not Sanford. Surgeons’ self-reported *knowledge* (mean score for domain in online survey = 3.95, SD *= *0.69) of guidelines and *skills* (mean *= *3.77; SD *= *0.69) related to guideline implementation was good. Surgeons had confidence in their ability to implement guidelines into practice (*belief in capabilities*, mean *= *3.59; SD *= *0.62) with little worry or anxiety provoked by the thought of it (mean *= *2.17, SD *= *0.88). A barrier existed in relation to access; where hard copies of local policy did exist, some reported difficulties in gaining access to them (i.e. only a few copies available in the hospital). Most instead used electronic resources and sought information online.

**Figure 1.**
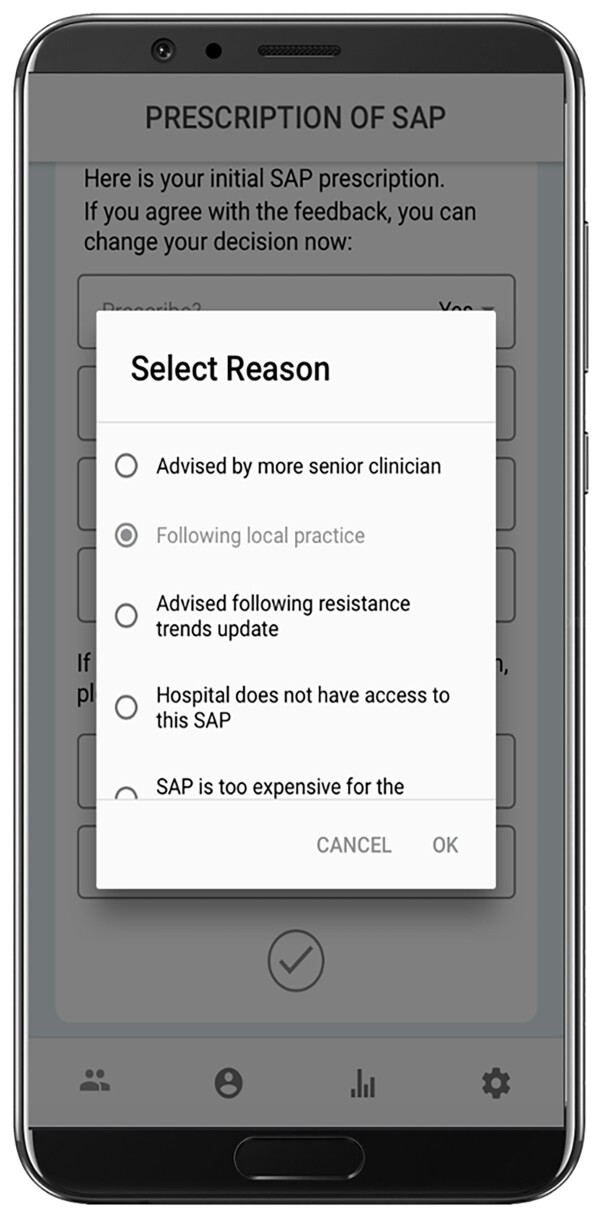
Screenshot of in-app data collection of reasons for diverting from WHO and Sanford guidelines.

Intention to implement guidelines was strong (mean score for *intention* domain from online survey responses = 4.31, SD *= *0.71), however it was reported ‘general principles’ rather than written policy guided local practice within departments, with information on guideline use (i.e. which SAP, recommended dosage) based on ‘what has worked’ and passed from senior to junior staff through hands-on experience. ‘Following local policy and practice’ rather than guidelines was also reflected in the data collected by the GADSA app, appearing as the main reason surgeons chose to divert from recommendations (111 out of a total of 266 prescriptions; 41.7%; see Table 3 and Figure 1 for a screenshot of how these appeared in the GADSA app).

**Table 3.** Reasons given for diversion from published guidance as recorded in the GASDA app

Reason given by surgeon for diverting from WHO and Sanford guidelines	Frequency	
	*n*	%
Following local practice	111	41.7
Patient is at high risk for developing SSI	59	22.2
Patient’s environment requires prolonged dose	56	21.0
Previous experience with this procedure	20	7.5
Advised by a more senior clinician	7	2.6
Patient has complications requiring prolonged dose	6	2.3
Advised by a colleague	2	0.8
Other reason (not listed)	2	0.8
Patient transferred to accident and emergency	1	0.4
Patient has an allergy	1	0.4
Hospital does not have access to recommended SAP	1	0.4
Total	266	100

From a total of 266 prescriptions diverting from guidelines, out of 343 prescription decisions recorded by the app.

Most believed following guidelines would reduce rates of SSIs (mean score for *beliefs about consequences* domain from online survey = 4.32, SD *= *0.80). Surgeons believed that use of guidelines for SAP prescription was key to slowing spread of AMR across Nigeria and essential for increasing transparency of practice across departments and specialisms at hospital level. Most, however, felt reluctant to change practice unless the patient required it or there was good evidence (supported by research in the Nigerian context) that this would lead to improved health outcomes. Surgeons were unsure whether guidelines could be effectively implemented in Nigeria or more importantly, whether it would be appropriate to do so given they reflect more accurately high-to-middle-income countries. Current guidelines strongly advise against the prolongation of SAP beyond 24 h post-operation, however, almost all surgeons expressed strong concerns related to risk of patients developing SSIs either in the hospital or after they return home with little or no option of follow-up visits to the hospital due to distance or cost.

Concerns about the risk of infection from the patient’s home environment or doubts the patient would continue to carry out adequate self-care as advised (e.g. regularly cleaning wounds) saw SAP prescribed as a preventive, with dosage prolonged beyond the recommendation (in some cases, for more than 10 days as standard practice). Data collected by the GADSA app cited ‘patient at high risk for SSI’ for 22.2% (or 59 out of a total of 266) of prescriptions diverting from guideline recommendations and ‘patient’s environment requires prolonged dose’ for 21.0% (or 56 out of 266) of prescriptions.

Environmental context and resources was a key barrier (mean scores for this domain in data collected by the online survey = 2.47, SD *= *0.70). Recommended SAPs were unavailable from the local hospital pharmacy or too expensive for patients to afford. Purchase decisions about specific brands of SAP and availability from the hospital pharmacy were made by senior management teams with decisions influenced by pharmaceutical advertising. Surgeons relied on specialist knowledge and recommendations of pharmacists dispensing the SAPs. Only one surgeon indicated this as a reason for diversion from guideline recommendations in data collected by the GADSA app (0.4% of a total 266 prescriptions).

Social context and behavioural norms surrounding prescription of antibiotics was also evident across all three types of data. Patients ‘expected’ to receive a prescription for a surgical procedure; a view likely exacerbated by the privatized medical insurance model in Nigeria where healthcare is considered a product and a service. If the surgeon did not prescribe, there was a perceived risk that the patient would seek antibiotics elsewhere and potentially from unregulated sources posing serious risk to their health. Some explained that lack of regulation surrounding access and sale of antibiotics in Nigeria meant that they could never be sure of the treatment patients had received prior to arriving at the hospital.

Surgeons’ beliefs relating to their social, professional role and identity also had a role in their implementation of guidelines (mean *= *3.71; SD *= *0.81). Decision-making in surgery is dynamic, requiring effective collaboration between the clinical team (e.g. junior doctors, nurses, pharmacists). Almost all considered guidelines to be ‘recommendations’ rather than checklists for practice with little or no consequences attached, either regulatory or socially, for those choosing not to follow them (mean score for domain of *reinforcement = *3.28, SD *= *0.82).

Professional experience and the collaborative environment was seen to play an equal, if not more important role, in determining ones prescribing decisions (‘previous experience with procedure’ was recorded in 7.5% or 20 of 266 prescriptions, ‘advised by senior clinician’ in 2.6% or 7 of the 266 prescriptions and ‘advised by a colleague’ in 0.8% or 2 of the 266 prescriptions diverting from guideline recommendations, in the GADSA app). Some considered guidelines more relevant for junior staff building up their knowledge rather than more senior surgeons with more experience.

## Discussion

Acknowledging that our pilot work reported here represents a snapshot from professionals at three hospital sites, our findings do align with those of similar studies.^11,12,13^ Professionals report a general awareness of prescribing guidelines. Most intend to apply guidance but report local environment (e.g. concerns related to the cleanliness of the operating, hospital and recovery environment, experiencing difficulties following up patients leaving hospital after their procedure) as a key barrier. Single-dose SAPs are considered impractical and routinely avoided for fear of patients developing infections post-operatively. Despite a relatively small sample of surgeons (*N *= 20) participating in Phase 3 and recording prescription at the point of care into the GADSA app, we saw first-hand the impact that these beliefs have on behaviour. For example, professionals defaulting to local practices not recommended by guidelines (e.g. prescribing SAP as a preventative and prolonging dose past the 24 h window). There are few consequences associated with not implementing guidelines (e.g. punishment from senior clinicians or management).

Based on these findings, we propose the following four approaches to support implementation of prescribing guidelines in Nigeria: (i) adapting international guidelines for local context; (ii) upskilling and empowering professionals to make informed decisions; (iii) enabling guideline implementation through mobile decision support tools; and (iv) integration with wider infection control policy.

### Adapting international guidelines for local context

Professionals diverted from guidelines because they knew they would not have access to the recommended SAP through their local hospital pharmacy. International guidelines generally reflect the regions from which data are collected and may not be appropriate for the local context. Recommended SAPs need to be based on local antibiotic susceptibility and resistance patterns. A point prevalence study of four tertiary hospitals in Nigeria showed that compliance with institutional guidelines for SAP was only 4.1% amongst surgeons.^28^ Further research led by those working within the country is needed as they are best placed to understand the local context and application. Guidelines directly informed by local research, local resistance patterns and prescription data would boost surgeon’s confidence and trust and would most likely significantly increase rates of implementation as a result. Learning where and when SAP substitutions are taking place will be vital for understanding how future guidelines can be adapted for low-to-middle-income countries and where recommendations for equivalent, yet cheaper or more readily available SAPs should be included.

### Upskilling and empowering professionals to make informed decisions

Professionals need to know how to adapt and apply guidelines to suit the requirements of individual patients. They should feel empowered and have confidence that they are implementing best practice in line with international standards whilst at the same time maintaining safety of their patients. This requires awareness raising of the consequences resulting from prescription outside of guideline recommendations (both for the local community and within the wider AMR context), retraining for clinicians on transmission of pathogens and how prophylaxis works including the limitations of antibiotics, knowledge building of the recommendations set out in guidelines and training provision on how they can be adapted to context.

### Enabling guideline implementation through mobile decision-support tools

The quality of training for medical trainees could be improved to build knowledge about guideline implementation. To foster longer-term behaviour change, it is essential that support should continue after training has been completed. Mobile technologies (i.e. smartphones, tablets) are fast becoming part of the standard toolkit available for use by healthcare professionals. As internet connectivity continues to improve globally and costs for mobile data become more affordable, a greater number of prescribing clinicians will benefit from having access to the latest evidence and best practice delivered straight to their pocket. Mobile decision-support tools, such as the GADSA app, that integrate information into one touchpoint and enable adaptation to local context will be an essential resource moving forward and one that will enable continued professional development and accurate implementation of guideline recommendations.

### Integration with wider infection control policy

Finally, guideline implementation is one element of a much wider and strongly interlinked infection control context. Reservations about cleanliness ultimately led clinicians to divert from guideline recommendations including prolonging prescription of SAP as a preventative for infection. Reviewing existing infection control policies and practice and putting in place measures to improve the clinical environment should therefore go hand-in-hand with any intervention to increase guideline implementation, as will be highlighted by another complementary study by C. E. Wood, S. Wiseman, F. Ncube, A. Molnar and P. Kostkova (unpublished results), ‘ICAN meets iNRIC: information needs of African IP&C professionals in the era of Internet and COVID – what has changed from 2015 to 2020’.

## Data sharing

Data from this research are available on request from the corresponding author.
